# Erratum: Morphological and Molecular Characterization of Oscheius saproxylicus sp. n. (Rhabditida, Rhabditidae) From Decaying Wood in Spain, With New Insights into the Phylogeny of the Genus and a Revision of its Taxonomy. DOI: 10.21307/jofnem-2019-053. Vol. 51

**DOI:** 10.21307/jofnem-2020-075

**Published:** 2020-07-27

Key to species identification

1a– Body length very long, 6 mm *latus*

1b – Body length shorter, less than 3.5 mm^ 2^

2a – Stoma barrel-shaped or tubular; bursa peloderan (unknown in O*. tereticorpus* and *O. saproxylicus* sp. n.) 3

2b – Stoma tubular; bursa leptoderan or pseudopeloderan 16

3a – Each spicule visibly with different size *sechellensis*

3b – Both spicules with similar size 4

4a – Female rectum scarcely longer than the anal diameter 5

4b – Female rectum ca. 2 to 3 times longer than the anal diameter 6

5a – Body length less than 750 µm; spicules 29 µm long *debilicauda*

5b – Body length more than 750 µm; spicules 32 to 48 µm long *zarinae*

6a – Female tail short conoid (*c*′ < 4) 7

6b – Female tail longer, elongate (*c*′ > 4, rarely 3) 9

7a – Lip region slightly offset by depression; female rectum longer, 3 times anal body diam. *dolichura*

7b – Lip region not offset; female rectum shorter, 2 times anal body diam. 8

8a – Males as frequent as females; spicules longer, 35 µm, exceeding the GP1 *janeti*

8b – Males very rare; spicules shorter, 22 to 30 µm, not reaching the GP1 *dux*

9a – Lip region visibly narrower than adjacent part of body; spicules longer, more than 40 µm 10

9b – Lip region equal or wider than adjacent part of body; spicules shorter, less than 30 µm (unknown in *O. tereticorpus* and *O. saproxylicus* sp. n.) 11

10a – Male body more than 1mm long; spicules longer, 41 to 48 µm *dolichuroides*

10b – Male body less than 1 mm long; spicules shorter, 50 to 52 µm *karachiensis*

11a – Gymnostom anteriorly narrower, with convex walls 12

11b – Gymnostom with parallel walls 14

12a – Lip region offset by constriction; metacorpus slightly swollen *pseudodolichura*

12b – Lip region not offset or slightly offset by depression; metacorpus not swollen 13

13a – Lip region slightly offset by depression; gymnostom as long as promesostegostom; pharynx with metacorpus with sclerotized walls, valves-like; spermatheca differentiated in a sac *tereticorpus*

13b – Lip region not offset; gymnostom slightly shorter than promesostegostom; pharynx with metacorpus without sclerotized walls; spermatheca not differentiated in a sac *saproxylicus* sp. n.

14a – Lip region slightly offset; female rectum ca. two times longer than ABW; GP1 very anterior, outside of the range of the spicules *guentheri*

14b – Lip region not offset; female rectum ca. three times longer than ABW; GP1 at spicules level 15

15a – Body length slightly larger (584-801 µm long); neck slightly shorter relative to the body length (*b* = 4.4-6.0); female tail slightly shorter (63-81 µm, *c* = 8.6–11.8, *c*′ = 3.5-5.0) *onirici*

15b – Body length slightly smaller (505-691 µm long); neck slightly longer relative to the body length (*b* = 3.9-4.9); female tail slightly longer (70-95 µm, *c* = 6.2-8.5, *c*′ = 4.2-6.4) *tipulae*

16a – Female rectum ca. as long or slightly longer than anal body width 17

16b – Female rectum longer than body width 22

17a – GP1 very reduced 18

17b – All GPs with similar size 20

18a – Spicules distally straight *wohlgemuthi*

18b – Spicules distally slightly hook-like 19

19a – Female body less than 1.7 mm long *cynodonti*

19b – Female body more than 1.8 mm long *rupraekramae*

20a – Female stoma 21 to 28 µm long *colombianus*

20b – Female stoma 12 to 20 µm long 21

21a – Female body 0.9 to 1.3 mm long; female tail more slender (*c*′ = 4.6-5.9) *magbooli*

21b – Female body 1.3 to 1.8 mm long; female tail shorter (*c*′ = 2.9-4.2) *esculentus*

22a – Stomatal tube shorter, ca. 1.0 to 2.0 times longer than wide 23

22b – Stomatal tube longer, ca. 2.5 to 3.0 times longer than wide 30

23a – Cheilostom as long as stomatal tube length *insectivorus*

23b – Cheilostom one third of the stomatal tube length 24

24a – GP1 very separated from GP2, and GP2-3 very close *caulleryi*

24b – GP1-2 distance similar or slightly more than GP2-3 distance 25

25a – Spicules with ventral bent tip 26

25b – Spicules with thin tip, crochet needle-like 28

26a – Spicules 43 to 52 µm, with thin tip; GP1-2 distance similar to GP2-3 distance *niazii*

26b – Spicules 50 to 62 µm, with thick tip; GP1-2 distance slightly more than GP2-3 distance 27

27a – Male tail shorter (20-32 µm long) *cobbi*

27b – Male tail longer (38-45 µm long) *siddiqii*

28a – Female rectum ca. 1.5 times longer than ABW; spicules shorter, 34 to 44 µm long *necromenus*

28b – Female rectum ca. 2.0 to 2.5 times longer than ABW; spicules longer, 45 to 70 µm long 29

29a – Spicules shorter (45-51 µm long) *andrassyi*

29b – Spicules longer (57-70 µm long) *citri*

30a – Spicules ventrad curved, as long as anal body width *esperacensis*

30b – Spicules almost straight, longer than anal body width 31

31a – Spicules ca. 1.5 times longer than the gubernaculum *myriophilus*

31b – Spicules ca. 2–3 times longer than the gubernaculum 32

32a – Spicules with manubrium longer than wide 33

32b – Spicules with manubrium as long as wide 35

33a – Lip region higher, twice wider than high *lucianii*

33b – Lip region lower, three times wider than high 34

34a – Stoma shorter, 9 to 10 µm; female tail shorter, (*c*′ = 3.1-4.8, rarely longer) *chongmingensis*

34b – Stoma longer, 13 to 18 µm; female tail longer, (*c*′ = 5.0-6.6) *indicus*

35a – Female rectum ca. 3.0 to 4.5 times longer than anal body width *shamimi*

35b – Female rectum ca. 1.5 to 2.0 times longer than anal body width 36

36a – Female tail shorter, *c*′ = 2.2 to 3.1 *rugaoensis*

36b – Female tail longer, *c*′ = 3.2 to 6.7 37

37a – Male tail with mucro shorter than gubernaculum; spicules with conoid manubrium *nadarajani*

37b – Male tail with mucro longer than gubernaculum; spicules with rounded manubrium *carolinensis*

